# Identification of Novel Inhibitors for Tobacco Mosaic Virus Infection in Solanaceae Plants

**DOI:** 10.1155/2015/198214

**Published:** 2015-10-18

**Authors:** Archana Prabahar, Subashini Swaminathan, Arul Loganathan, Ramalingam Jegadeesan

**Affiliations:** ^1^Data Mining & Text Mining Laboratory, Department of Bioinformatics, School of Life Sciences, Bharathiar University, Coimbatore, Tamilnadu 641046, India; ^2^Department of Plant Molecular Biology & Bioinformatics, Centre for Plant Molecular Biology & Biotechnology, Tamil Nadu Agricultural University, Coimbatore, Tamilnadu 641003, India

## Abstract

*Tobacco mosaic virus* (TMV) infects several crops of economic importance (e.g., tomato) and remains as one of the major concerns to the farmers. TMV enters the host cell and produces the capping enzyme RNA polymerase. The viral genome replicates further to produce multiple mRNAs which encodes several proteins, including the coat protein and an RNA-dependent RNA polymerase (RdRp), as well as the movement protein. TMV replicase domain was chosen for the virtual screening studies against small molecules derived from ligand databases such as PubChem and ChemBank. Catalytic sites of the RdRp domain were identified and subjected to docking analysis with screened ligands derived from virtual screening LigandFit. Small molecules that interact with the target molecule at the catalytic domain region amino acids, GDD, were chosen as the best inhibitors for controlling the TMV replicase activity.

## 1. Introduction

TMV infects tobacco and other members in the Solanaceae family which includes economically important plants like tobacco, tomato, potato, and pepper [1]. Tobacco mosaic virus has a rod-like appearance [2]. TMV is the type member of the genus* Tobamovirus* and has been studied extensively for its ability to replicate and induce host disease or resistance responses [3, 4]. Plants infected with TMV may have many symptoms including patches of light and normal green pigment on the leaves, chlorosis, dwarfing, blistering of the leaves, and damage to the fruit [5]. Even though TMV is able to infect many species and spread quickly but, the virus rarely kills the host. Virus-infected plants often display developmental abnormalities that include stunting, leaf curling, and the loss of apical dominance [6].

TMV is a positive-stranded RNA virus that encodes at least four proteins [3]. The two open reading frames (ORF) encode 126- and 183-kDa replicase proteins, where larger codon is produced through an amber stop codon [7]. Homology studies shows that the 126-kDa ORF protein encodes a methyl transferase domain (MT) involved in viral RNA capping and a helicase domain (HEL) involved in double-stranded RNA unwinding [8]. The read-through portion of the 183-kDa ORF encodes the RNA-dependent RNA polymerase domain (POL), a 30-kDa protein required for cell-to-cell movement and the 17.5-kDa capsid proteins are produced from mRNAs in the subgenomic region [9, 10].

The 183-kDa protein has amino acids motifs that are characteristic of RNA-dependent RNA polymerases (RdRp) and provides the catalytic activity for the synthesis of TMV RNA. While there has been no evidence for polymerase activity* in vitro*, TMV replicase complexes isolated from infected tissue have been shown to possess RdRp dependent polymerase activity [11]. The RdRp (replicase) mediates the replication of tobacco mosaic virus (TMV) [12].

The poliovirus RNA-dependent RNA polymerase, 3Dpol, is known to share a region of sequence homology with all RNA polymerases centered at the GDD amino acid motif. The two aspartic acids have been postulated to be involved in the catalytic activity and metal ion coordination of the enzyme [13].

**Figure 1:**
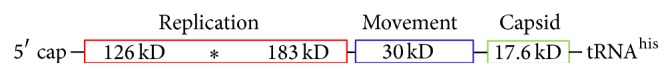
Genome with protein coding.

To cause a disease, a virus must be capable of replicating in host cells, moving into surrounding cells, and then progressing systematically throughout the plant. It is generally accepted that plant viruses traverse the cell wall through plasmodesmata (Pd), membrane-bound tunnels that interconnect the cytoplasm of neighboring cells. Although TMV MP is necessary for cell-to-cell spread of viral infection [14], replicase is also involved and remains as one of the key components necessary for cell-to-cell movement of TMV [15]. Our studies are based on the suggestion that replicase is part of a virus movement complex that contains all essential components necessary for virus replication (vRNA, replicase, and MP) [16]. Protein coding genome location of the TMV is shown in Figure 1. The nature of the infectious entity that moves to and through plasmodesmata is not known, although it was postulated that spread occurs as ribonucleoproteins complexes comprising the MP and TMV-RNA [17].

To control the most destructive TMV infection in plants, several plant transformation studies have been carried out. Replicase-mediated transgenic resistance was first discovered in 1990 by Golemboski et al. [18]. One of the recent studies is based on targeting the RNA-dependent silencing pathway through posttranscriptional gene silencing (PTGS), a mechanism through which plants protect themselves from viral infection. Inhibition of TMV in tobacco was demonstrated through RNA expression encoding the methyltransferase domain of TMV replicase and further RT-PCR analysis [19].

**Figure 2:**
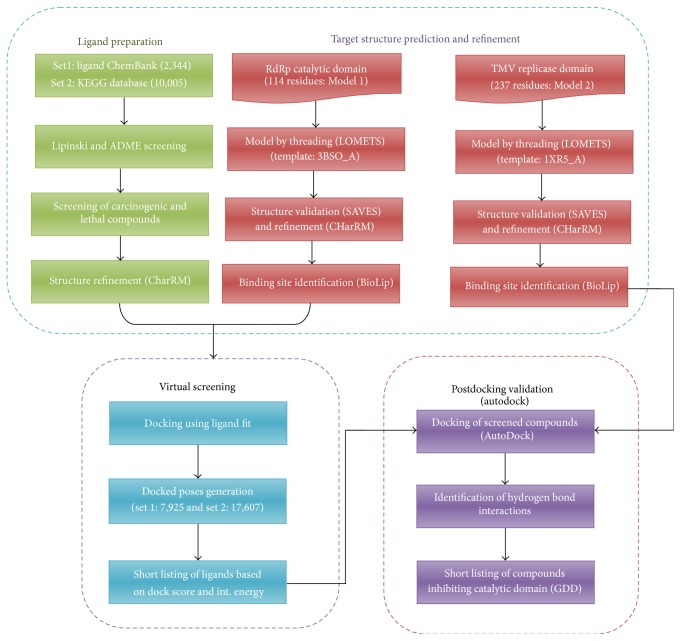
Workflow of TMV replicase inhibition study.

In this study, we chose RdRp domain as the target to control the TMV infection in plants. Virtual screening is performed to identify the potential ligand that could inhibit the catalytic domain of TMV replicase. We examined the ability of inhibitory effect of these small molecules through postdocking studies after virtual screening to identify the small molecules that target a much larger RdRp domain involving catalytic region amino acid residues (GDD). Our results demonstrated that top six ligands outperform the analysis and hence inhibit the TMV replicase catalytic domain activity. A schematic representation of various steps involved in the inhibitory action against TMV replicase is shown in Figure 2. Hence, the current study aims to exemplify that the small molecules would inhibit the activity of the TMV replicase at the catalytic sites and prevent the spread of TMV infection in several plants of Solanaceae family. To our knowledge, this is the first virtual screening study to perform an inhibitory action against RdRp catalytic domain in TMV replicase.

## 2. Materials and Methods

### 2.1. Selection of TMV Replicase Protein

The protein sequence of TMV replicase gene was retrieved from ExPASy Proteomics Server (http://expasy.org/sprot/). The sequence id** P03586** (RDRP_TMV) of tobacco mosaic virus (strain vulgare) was taken for this study. In the first case, RdRp catalytic domain region of** P03586** (1380–1493) which is 114 residues in length was selected. Later in this study, the whole RdRp domain region was selected (1380–1616) based on the PSI-BLAST result, which was 237 residues in length.

### 2.2. Modeling of Catalytic Domain Using LOMETS

Catalytic domain region was modeled using threading technique due to the lack of 3D crystal structure templates with sequence similarity greater than 30%. LOMETS (Local Meta-Threading-Server) server (http://zhanglab.ccmb.med.umich.edu/LOMETS/), an online web service for protein structure prediction, was utilized. It generated protein structures by ranking and selecting models from 8 state-of-the-art threading programs and generated 3D models by collecting high-scoring target-to-template alignments from 9 locally installed threading programs (FFAS-3D, HHsearch, MUSTER, pGenTHREADER, PPAS, PRC, PROSPECT2, SP3, and SPARKS-X). Structure of Model 1 and Model 2 generated by LOMETS based on the rank was downloaded for the study [20].

### 2.3. Structure Validation Using SAVS

Model thus generated was assessed using SAVES (*Structural Analysis and Verification Server*), the Metaserver for analyzing and validating protein structures (http://nihserver.mbi.ucla.edu/SAVES/). Using Procheck, the stereochemical quality of a protein structure was assessed by analyzing residue-by-residue geometry and overall structure geometry. Ramachandran plot is further analyzed to study the percentage of residues that lie in the different regions such as favorable, allowed and disallowed regions.

### 2.4. Active Site Identification

From the literature study, the poliovirus RNA-dependent RNA polymerase, 3Dpol, was known to share a region of sequence homology with all RNA polymerases centered at the GDD amino acid motif. The two aspartic acids have been postulated to be involved in the catalytic activity and metal ion coordination of the enzyme [21]. 3D models were built based on multiple-threading alignments by LOMETS [20] and iterative TASSER [22] assembly simulations; function insights were then derived by matching the predicted models with protein function database named BioLip. BioLiP is a curated database for identification of ligand-protein binding interactions. In order to annotate the function of uncharacterized proteins, a new algorithm COACH is used to predict ligand-binding sites. Predicted ligand-binding sites were sorted out based on the confidence score.

### 2.5. Selection of Small Molecules Dataset

The small molecule datasets chosen for this study include KEGG and ChemBank datasets from ligand info database, which is a comprehensive collection of publicly available databases. ChemBank ligand entries and KEGG ligand entries were downloaded from Ligand Info (http://ligand.info/) in SDF format. The ChemBank subset and KEGG subset have 2344 (ligand dataset 1) and 10,005 (ligand dataset 2) entries of ligand, respectively, and were used for virtual screening and docking studies by using Discovery Studio/LigandFit program (version 1.7, Accelrys Software Inc.). The compound records contain calculated three-dimensional coordinates with bioactivity information and FDA approval status for certain biomolecules.

### 2.6. Structure Refinement Using Discovery Studio

#### 2.6.1. CHARMm Force Field

The modeled structures were subjected to CHARMm (Chemistry at HARvard Macromolecular Mechanics) force field for structure refinement. This includes removal of water molecules and addition of missing atoms and introduction of appropriate charges, such as protonation of amino groups. The modeled structure was also checked for valency, missing hydrogen, and any structural disorders like connectivity and bond orders. Further hydrogen atoms were added and CHARMm force field was applied for energy minimization. Energy minimization was performed for all compounds using CHARMm force field with an energy gradient of 0.001 kcal/mol/A°. Force fields also include energy components such as bond, angle, and torsion parameters, along with atom charges, radii, and van der Waals energy minima to represent nonbonded interactions and further simulation of molecular parameters. Using Discovery Studio (DS), force fields were applied using the functionality provided in the force field tool panel. Missing parameters were also edited using the force field Window.

#### 2.6.2. Lipinski Rule and ADME Prediction

Molecular properties of the ligand subsets were inspected using Lipinski rule. The rule states that most “drug-like” molecules have log*P* ≤ 5, molecular weight ≤500, number of hydrogen bond acceptors ≤10, and number of hydrogen bond donors ≤5. Molecules violating more than one of these rules may have problems with bioavailability. The rule is called “Rule of 5,” because the border values are 5, 500, 2*∗*5, and 5 [23, 24]. It is tested using Molinspiration tool (http://www.molinspiration.com/). Molecules satisfying the Lipinski rule were used for further studies.

### 2.7. ADME Prediction

Most of the drug molecules fail during clinical trials due to poor ADME (absorption, distribution, metabolism, and excretion) properties. Prediction of ADME properties prior to expensive experimental procedures would aid in the selection of successful candidates. Use of these candidates which succeed ADME properties against TMV would not cause any lethal effects during consumption of crop yields by human. ADME properties are predicted using Qikprop tool available in the Schrodinger software. ADME properties are thus calculated for ligand dataset 1 and ligand dataset 2.

### 2.8. Virtual Screening Using LigandFit

The LigandFit docking algorithm is an interactive procedure in which random ligand conformations were generated a specified number of times, NmaxTrial. DS LigandFit aids in docking ligands into the binding site of receptors using shape-based searching and Monte Carlo sampling of ligands. Parameters can be customized and DS LigandFit can be parallelized in multicore machines or clusters for virtual high-throughput screening. The internal energy of the ligand is computed when using the force field version of dock score. Dock score is computed based on

(1) Dock Score forcefield=−ligandreceptor interaction energy+ligand internal energy.

In this study both dock score and internal energy of the compounds are considered.

### 2.9. Short Listing Based on Dock Score

After virtual screening of small molecules using LigandFit, molecules that have interacted with the macromolecule were chosen for the docking study. Top scoring molecules were shortlisted based on the dock score and internal energy.

### 2.10. Postdocking Confirmation Studies Using AutoDock

The Graphical User Interface program “AutoDock Tools” was used to prepare, run, and analyze the docking simulations. Kollman united atom charges, solvation parameters, and polar hydrogens were added into the receptor PDB file for the preparation of protein in docking simulation. AutoDock [25–27] requires precalculated grid maps, one for each atom type present in the flexible molecules being docked, and it stores the potential energy arising from the interaction with rigid macromolecules. This grid must surround the region of interest in the rigid macromolecule. The grid box size was set at 60, 60, and 60 A° (*x*, *y*, and *z*) to include all the amino acid residues that were present in rigid macromolecules. AutoGrid 4.0 Program, supplied with AutoDock 4.0, was used to produce grid maps. The spacing between grid points was 0.375 angstroms. The Lamarckian Genetic Algorithm (LGA) 23 was chosen to search for the best conformers. During the docking process, a maximum of 10 conformers was considered. The population size was set to 150 and the individuals were initialized randomly. Maximum number of energy evaluations were set to 500000, maximum number of generations were set to 1000, maximum number of top individuals that automatically survived were set to 1, mutation rate is 0.02, crossover rate is 0.8, and step sizes were 0.2 Å for translations, 5.0° for quaternions, and 5.0° for torsions. Cluster tolerance is 0.5 Å, external grid energy is 1000.0, max initial energy is 0.0, max number of retries are 10000, and 10 LGA runs were performed. AutoDock was compiled and run under Windows XP operating system. AutoDock results were analyzed to study the interactions and the binding energy of the docked structures.

## 3. Results and Discussion

### 3.1. Modeling of RdRp Catalytic Domain

Catalytic domain was modeled by threading technique using LOMETS (Local Meta-Threading-Server) server. RdRp catalytic domain,** P03586** [1380–1493], which is 114 residues in length was thus modeled. The template used by LOMETS was 3BSO_A with a *Z*-score of 33% with high confidence. Model thus generated (Model 1) has been used for virtual screening studies. Later in this study, PSI-BLAST was performed. As a result, RDRP domain region was extended further** P03586** [1380–1616] to 237 residues in length. Model 2 was predicted using LOMETS threading sever. The template used by LOMETS was 1xr5_A with a *Z*-score of 38% and with high confidence. This model was used in for postvirtual screening studies using docking (AutoDock).

### 3.2. Model Validation

**Figure 3:**
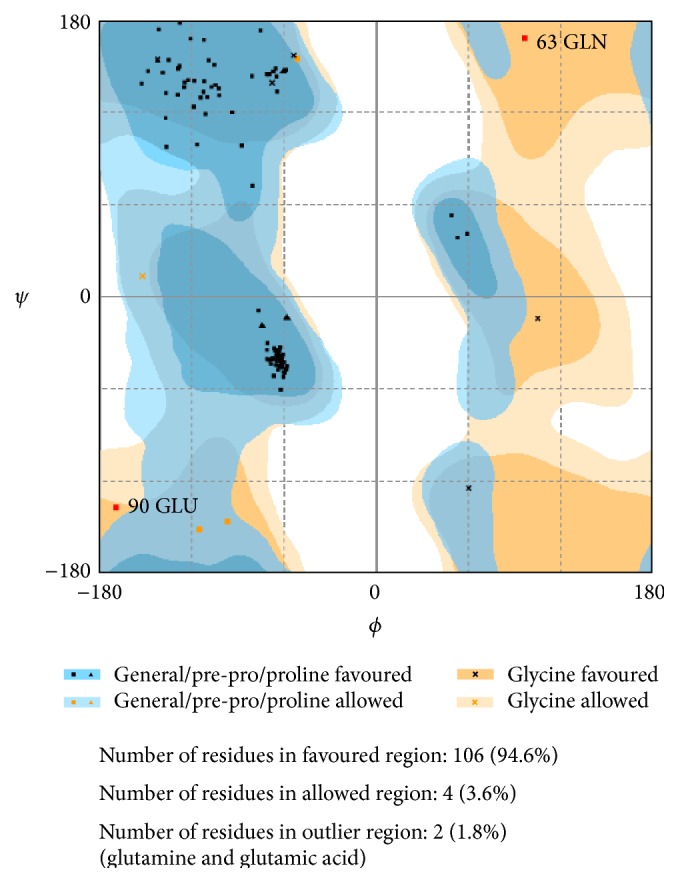
Ramachandran plot of Model 1 (**P03586** (1380–1493)).

**Figure 4:**
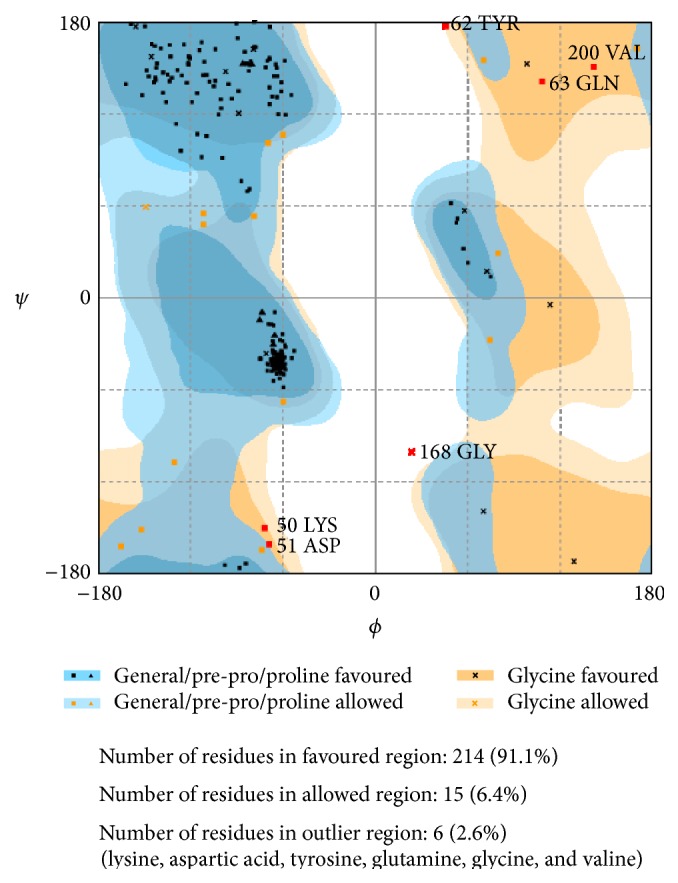
Ramachandran plot of Model 2 (**P03586** (1380–1616)).

Initial model (Model 1) was validated using Procheck, and the residues in most favoured regions (Ramachandran plot) were found to be 94.6%. Later using Procheck, the longer model (Model 2) thus generated was validated and the residues in most favoured regions (Ramachandran plot) were found to be 91.1%. The Ramachandran plot of Model 1 and Model 2 are shown in Figures 3 and 4, respectively.

### 3.3. Active Site Identification

**Figure 5:**
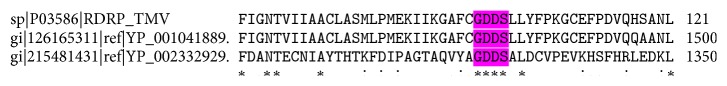
CLUSTALW alignment, GDDS motif region.

**Figure 6:**
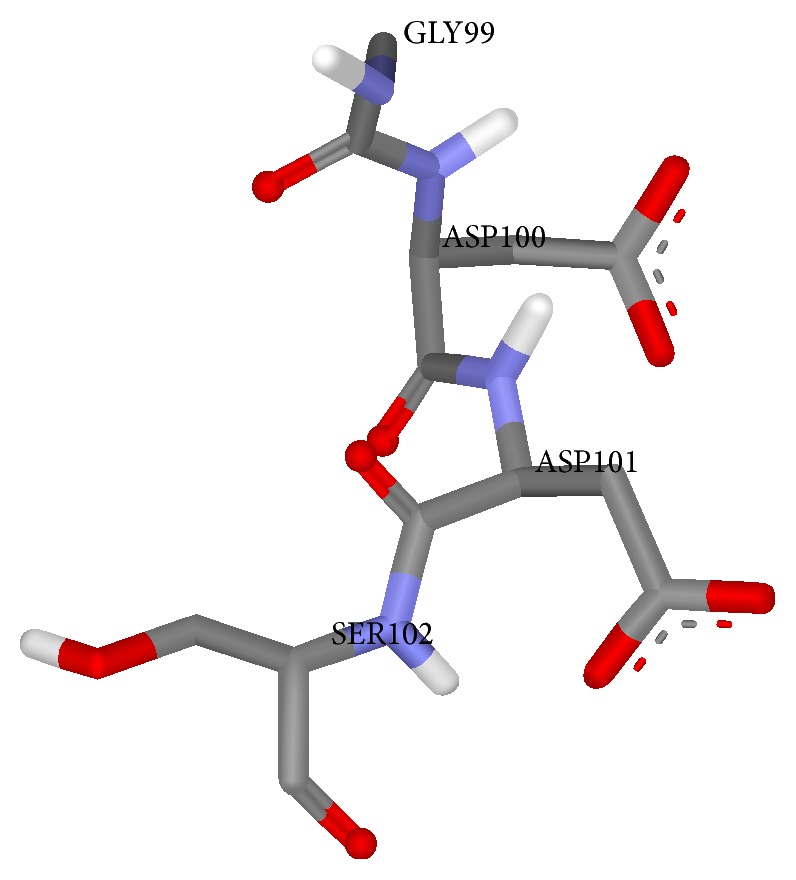
Structure of GDD motif region in RNA-dependent RNA polymerase.

To verify the active site regions in TMV replicase, clustalw alignment is done using the sequences of TMV replicase, Rehmannia mosaic virus, and Potato virus X (strain X3). The conserved domain is found at GDDS amino acid motif in all these sequences. The clustalw alignment of GDDS motif is shown in Figure 5. The binding site residues in Model 1 were found to be ASP7, ILE8, SER9, LYS10, TYR11, ASP12, SER66, THR71, ASN75, and ASP100. The binding site residues in Model 2 were found to be ASP7, ILE8, SER9, LYS10, TYR11, ASP12, ARG64, SER66, THR71, ASN75, GLY99, ASP100, ASP101, and LYS128. From these studies, GDD motif is chosen to be the active site region for RNA-dependent RNA polymerase domain of TMV replicase and the structure of this motif is shown in Figure 6.

### 3.4. Lipinski Rule and ADME Properties Prediction

Most important ADME properties such as Polarizability, Human Oral Absorption in GI, aqueous solubility, BB for brain/blood, serum protein binding Lipinski rule of 5 violations, and Jorgensen Rule of 3 violations of the final inhibitor molecules are tabulated in Table 1. Along with the compounds that do not follow Lipinski and ADME properties, certain small molecules were known to have carcinogenic and lethal properties. They are screened from the list and further eliminated from this study.

### 3.5. Virtual Screening Using LigandFit

Model 1 was chosen as the receptor for virtual screening studies and the small molecules of ChemBank dataset were used as the ligand set and hence virtual screening is performed. As a result, 7,925 docked poses were obtained after docking 2,344 small molecules. For the virtual screening study 2, Model 1 was chosen as the receptor and the small molecules of KEGG dataset were used as the input ligands. As a result, 17,607 docked poses were obtained after docking 10,005 small molecules.

### 3.6. AutoDock Docking Studies

Molecules were shortlisted based on the drug-like properties analyzed using pharmacological properties for further wet lab studies. These small molecules were subjected to docking studies using AutoDock 4.0 docking software, where Model 2 acts as the receptor molecule. After performing the docking studies, results were analyzed and the binding energy and the hydrogen bonding regions were identified. Small molecules were further shortlisted based on these properties. Small molecules forming hydrogen bonding at the active site region GDD are chosen for further studies.

**Figure 7:**
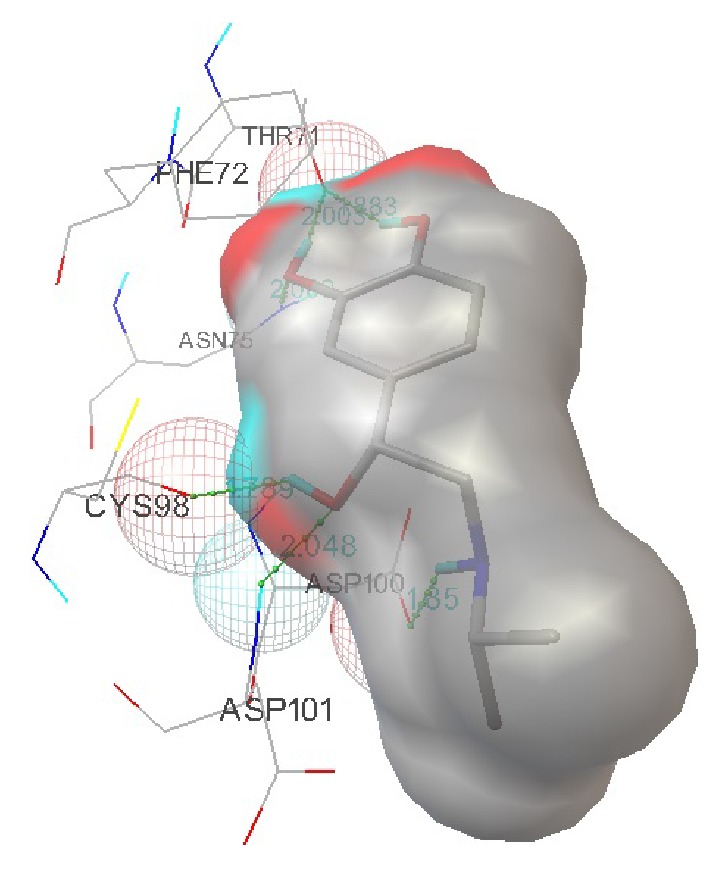
Representation of the docked model of TMV replicase with isoproterenol.

Shortlisted ligands were further screened based on their drug properties. Carcinogenic drugs were eliminated in this study. Ligands with similar properties were screened and those with higher binding affinity and more hydrogen bond interactions were chosen for the study. Based on all these properties five ligands were chosen as inhibitors against TMV and listed in Table 2. Among these five inhibitors isoproterenol was found to be the best inhibitor against TMV replicase with a higher binding affinity of −7.17 and a maximum of six hydrogen bond interactions of which two interactions were found at its active site regions. The docked interaction with the hydrogen bonds formed is shown in Figure 7. These results were shown in Table 2.

Vitamins are known to be activators in various plant defense mechanisms [28–30] and hence Riboflavin would remain as a potential inhibitor of TMV replicase with a binding energy of −5.17 forming four hydrogen bonds.

## 4. Conclusion

TMV infections cause significant damage to economically important crops such as tomato. There are approximately 450 species of pathogenic plant viruses and many are responsible for huge losses in crop production and quality in all parts of the world. After screening several small molecules from ChemBank and PubChem databases, we conclude that isoproterenol would be the potent inhibitor against TMV forming a maximum of six hydrogen bond interactions with binding affinity of −7.17 kcal/mol. Five more ligands were also shortlisted based on their inhibitory action against TMV. All these small molecules are available as drugs and can be prepared as a spray to treat TMV infections. These small molecules were prescreened for drug properties such as ADMET in order to test their drug-likeliness and bioavailability and prove that there is no harm upon consumption of end-products of the economically important crops by human.

## Figures and Tables

**Table 1:** ADME prediction.

Property	Molecules						Limits
	Albendazole	Atropine	Isoproterenol	Riboflavin	Neomycin	Ampicillin	
QP Polarizability	28.617 M	33.375 M	21.120 M	31.904 M	25.229 M	33.248 M	13.0/70.0
% Human Oral Absorption in GI (+−20%)	94	88	63	23	13	20	(<25% is poor)
Qual. Model for Human Oral Absorption	High	High	Medium	Low	Low	Low	(>80% is high)
QP log⁡*S* for aqueous solubility	−4.456	−2.465	−0.842	**0.915**	**1.427**	−1.506	(−6.5/0.5)
QP log BB for brain/blood	−0.816	0.040	−0.828	−2.746	−2.505	−1.092	(−3.0/1.2)
QP log⁡*K* hsa serum protein binding	0.221	0.122	−0.579	**−2.435**	**−2.933**	−0.936	(−1.5/1.5)
Lipinski rule of 5 violations	0	0	0	0	0	0	(Maximum is 4)
Jorgensen Rule of 3 violations	0	0	0	1	1	1	(Maximum is 3)

**Table 2:** Docking interactions with small molecules (hydrogen bond between target and ligands with its binding distance).

Ligand	Property	No hydrogen bonds	Binding energy (kcal/mol)	Hydrogen bond donor	Hydrogen bond acceptor	Hydrogen bond distance (Å)
Isoproterenol	Beta adrenergic agonist	6	−7.17	Lig::UNK1:H Lig::UNK1:H ASN75:HD21 **ASP101:HN:** Lig::UNK1:H Lig::UNK1:H	CYS98:O THR71:OG1 Lig::UNK1:O Lig::UNK1:O **ASP100:OD1** THR71:OG1	1.789 1.883 2.069 1.85 2.048 2.003

Riboflavin	Vitamin B2	4	−5.17	**ASP100:HN** Lig::UNK1:H Lig::UNK1:H Lig::UNK1:H	Lig::UNK1:O ASP12:OD1 **ASP100:OD2** **ASP100:OD2**	1.941 1.826 1.889 1.778

Atropine	Tropane alkaloid anticholinergic drug	2	−5.53	Lig::UNK1:H **ASP101:HN**	**ASP100:OD2** Lig::UNK1:HO	2.146 2.213

Albendazole	Benzimidazole compound	3	−5.39	Lig::UNK1:H LYS157:HZ3 Lig::UNK1:H	**ASP101:OD2** Lig::UNK1:O **ASP101:OD2**	1.829 1.956 1.94

Neomycin	Aminoglycoside antibiotic	4	−4.28	Lig:unk1:H: Lig:unk1:H: Lig:unk1:H: Lig:unk1:H:	**ASP100:OD2** **ASP101:0D2** **ASP100:0D1** ILE8:0	2.119 2.083 1.667 2.083

Ampicillin	Bacteriolytic, *β*-lactam antibiotic	3	−7.66	Ligand::unk1:H1 LYS157:HZ1 Ligand::unk1:H2	**ASP100:0D1** Ligand:UNK0:O **ASP100:OD2**	1.863 1.911 2.015
